# Orbital-hybridization-created optical excitations in Li_2_GeO_3_

**DOI:** 10.1038/s41598-021-84506-0

**Published:** 2021-03-02

**Authors:** Vo Khuong Dien, Hai Duong Pham, Ngoc Thanh Thuy Tran, Nguyen Thi Han, Thi My Duyen Huynh, Thi Dieu Hien Nguyen, Ming Fa-Lin

**Affiliations:** 1 Department of Physics, National Cheng Kung University, Tainan, 701 Taiwan; 2 Center of General Studies, National Kaohsiung University of Science and Technology, Kaohsiung, Taiwan; 3 Hierarchical Green-Energy Materials (Hi-GEM) Research Center, National Cheng Kung University, Tainan, 701 Taiwan; 4 Department of Chemistry, Thai Nguyen University of Education, 20 Luong Ngoc Quyen, Quang Trung, Thai Nguyen City, Thai Nguyen Province Vietnam

**Keywords:** Mathematics and computing, Nanoscience and technology, Optics and photonics, Physics

## Abstract

The three-dimensional ternary Li_2_GeO_3_ compound presents various unusual essential properties. The main features are thoroughly explored from the first-principles calculations. The concise pictures, the critical orbital hybridizations in Li–O and Ge–O bonds, are clearly examined through the optimal geometric structure, the atom-dominated electronic energy spectrum, the spatial charge densities, the atom and orbital-decomposed van Hove singularities, and the strong optical responses. The unusual optical transitions cover the red-shift optical gap, various frequency-dependent absorption structures and the most prominent plasmon mode in terms of the dielectric functions, energy loss functions, reflectance spectra, and absorption coefficients. Optical excitations, depending on the directions of electric polarization, are strongly affected by excitonic effects. The close combinations of electronic and optical properties can identify a significant orbital hybridization for each available excitation channel. The developed theoretical framework will be very useful in fully understanding the diverse phenomena of other emergent materials.

## Introduction

Materials that combine good optical characters with high electrochemical performances are of great technological interest^1–3^. They can be the central ingredient in the lithium-based batteries governing modern electronic devices and are used in electro-optic applications. Lithium Germanate (Li_2_GeO_3_) belongs to this class of materials. Indeed, the tetrahedral structure of Li–Ge–O compound exhibits a reliable ionic conductivity $\left( {5 \times 10^{ - 5} \,\Omega .{\text{cm}}^{ - 1} } \right)$^4^, in which the electronic conductivity is negligible with a high ionic transference number. This ternary compound shows wide cycling stability with a reserved charge capacity of 725 mAhg^−1^ after 300 cycles at 50 mAg^−1^^5^. As for the low-cost and safe batteries, the Li_2_GeO_3_ compound is suitable for the selection of anode/electrolyte in LIBs^5,6^. On the other hand, the good optical quality of Li_2_GeO_3_ has also been reported in the previous works^7–9^. The high optical transparency, the strong Stock shift of self-trap excitons, the polar orthorhombic symmetry in the crystal of Li_2_GeO_3_ strongly demonstrate that it is a remarkable interest material for piezoelectric, pyroelectric, and optoelectronic applications.

Although the high potential of Lithium Germanate compound in optoelectronic and battery applications is expected, the fundamental properties, such as electronic structure and optical properties of Li_2_GeO_3_ were not been completely comprehended. (i) Density functional theory (DFT) is a good starting point to understand the electronic properties of solid-state substances. However, it cannot correctly estimate the fundamental band gap of materials^10^, especially for large gap semiconductors or insulators. For example, the previous standard DFT calculation indicated the band gap of 3.8 eV for Li_2_GeO_3_^6^, it is not consistent with the optical measurement of Trukhin and his coworkers^7^. (ii) Up to now, the optical characters of Li_2_GeO_3_ have been detected by the photoluminescence measurements^7^. Other experiments or calculations on reflection, absorption coefficients, and energy loss functions to thoroughly understand the optical properties of Li_2_GeO_3_ are absent. Furthermore, the excitonic effects—the mutual interactions between excited electrons and holes, are usually observed in materials within suitable conditions, e.g., large band gap or low temperature. This electron–hole pair strongly influences on optical excitations and thus worthy to include in calculations. However, the theoretical studies to point out the effect of exciton on optical properties of Li_2_GeO_3_ have not achieved. (iii) The significant orbital hybridizations that survive in chemical bonds are rather complicated and responsible for the critical fundamental properties^11–13^. However, the investigations to identify such critical bonding mechanism in lithium-based materials are still rather limited. Furthermore, the systematic connection of the orbital hybridizations with the electronic properties, optical excitations and excitonic effects is a question that has not been answered up to now. The main reasons are the presence of large unit cell, non-uniform chemical environments, and thus, the extremely complicated orbital hybridizations in these ternary compounds^14^.

In this work, the theoretical framework, being based on the significant orbital hybridizations in chemical bonds^6,15,16^, is developed by examining the essential properties in the ternary lithium-based materials. This strategy is based on the accurate first-principles calculations on an optimal lattice symmetry with position-dependent chemical bondings, the atom-dominated band structure at different energy ranges, the spatial charge densities due to various orbitals, and the atom- and orbital-projected van Hove singularities related to orbital overlaps. The energy-decomposed single-/multi-orbital hybridizations will be utilized to account for the optical threshold frequency, a lot of prominent absorption structures, a very strong plasmon response in terms of the dielectric functions, energy loss functions, reflectance spectra, and absorption coefficients under the distinct electric polarizations, the excitonic states with rather strong energy binding. The current study is of paramount importance not only for fundamental physics but also for technical applications. Most predicted results in this work require high-resolution experimental examinations.

## Computational details

We used the Vienna Ab-initio Simulation Package (VASP) to perform the optimization of the structure and calculation the electronic and optical properties. For the ground states calculations, the Perdew–Burke–Ernzerhof (PBE) generalized gradient approximation^17^ was used for the exchange–correlation functional. Projector augmented wave (PAW) pseudopotentials are used to describe the electronic wave functions in the core region^18^. The cutoff energy for the expansion of the plane wave basis was set to 500 eV. The Brillouin zone was integrated with a special k-point mesh of 15 × 15 × 15 in the Monkhorst–Pack sampling technique^19^ for geometric optimization. The convergence condition is set to be 10^*–*8^ eV between two consecutive simulation steps, and all atoms were allowed to fully relax during geometric optimization until the Hellmann–Feynman force acting on each atom was smaller than 0.01 eV/Å.

Subsequent to the DFT results, the single-particle Green’s function and the screened Coulomb interactions (G0W0) approach^20^ using 250 eV energy cutoff for the response functions and the Brillouin zone was integrated with a special k-points mesh of 10 × 10 × 10 in the ${\Gamma }$-center sampling technique to obtain the corrected quasi-particle density of states and electronic band structure. The single-particle optical excitation of Li_2_GeO_3_ can be described by the Kubo formula^21^:

$$\epsilon_{2} \left( \omega \right) = \frac{{8\pi^{2} e^{2} }}{{\omega^{2} }}\mathop \sum \limits_{vc\textbf{k}} \left| {\textbf{e}.\left<v\textbf{k}{|}\textbf{v}{|}c\textbf{k}\right>} \right|^{2} \delta \left( {\omega -\left(E_{c\textbf{k}} - E_{v\textbf{k}}\right)} \right),$$

where the intensity of each excitation peak and the available transition channels is directly related to the velocity matrix element, $\left| {\textbf{e}.\left<v\textbf{k}{|}\textbf{v}{|}c\textbf{k}\right>} \right|^{2},$ and joined of density of states $\delta \left( {\omega -\left(E_{c\textbf{k}} - E_{v\textbf{k}}\right)} \right)$, respectively. Regarding the optical response beyond the independent particle approach, the electron–hole interaction was taken into account. The connection of the exciton energies ${\Omega }_{S}$ and corresponding electron–hole amplitude $\left| {\textbf{e}.\left<0{|}\textbf{v}{|}S\right>} \right|^{2}$ of the correlated electron–hole excitations S is obtained by solving the Bethe–Salpeter equation (BSE)^21^

$$\epsilon_{2} \left( \omega \right) = \frac{{8\pi^{2} e^{2} }}{{\omega^{2} }}\mathop \sum \limits_{{vc{\textbf{k}}}} \left| {\textbf{e}.\left<0{|}\textbf{v}{|}S\right>} \right|^{2} \delta \left( {\omega - {\Omega }_{s} } \right).$$

In which, the k-point sampling, energy cutoff, and number of bands setting the same as in the G0W0 calculation. In this calculation, the 20 highest occupied valence bands (VBs) and 8 lowest unoccupied conduction bands (CBs) are included as a basis for the excitonic states with a photon energy region from 0 to 25 eV. In addition, the broadening parameter *γ*, which arises from various de-excitation mechanisms, was set at 0.1 eV. All parameters in this study have been checked for convergence of the calculations.

## Results and discussions

**Figure 1:**
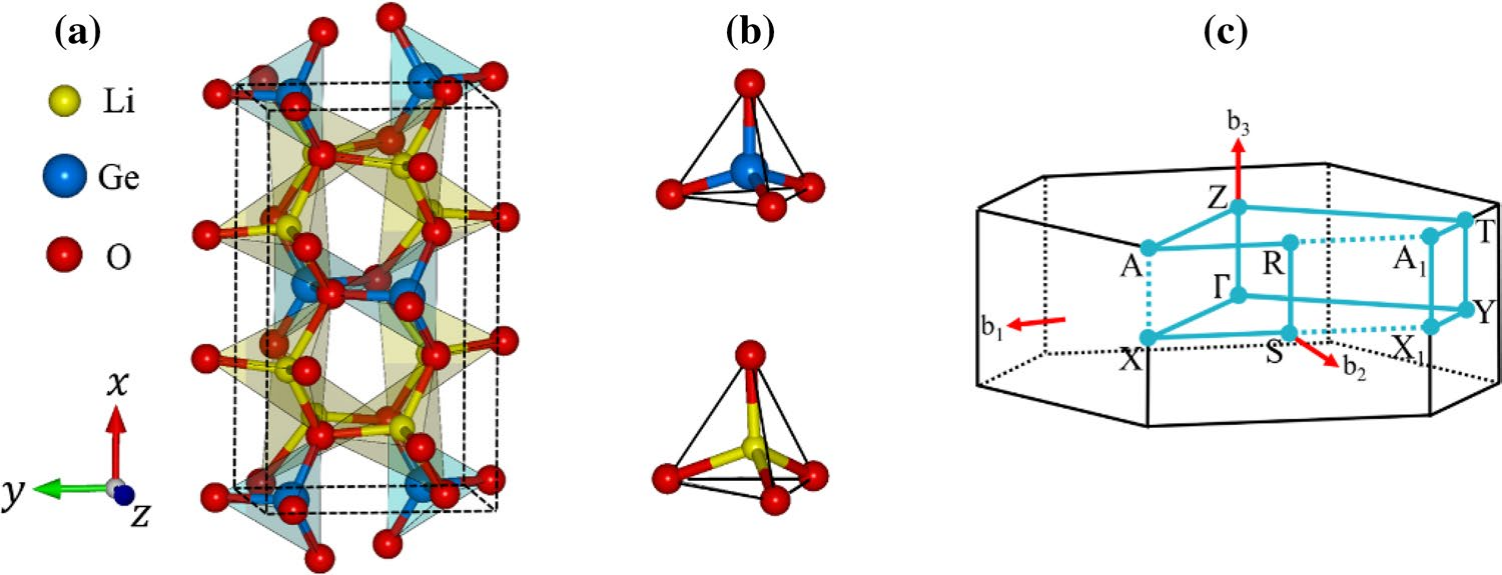
The optimal geometric structure of the Li_2_GeO_3_ ternary compound, (**b**) oxygen atoms around each Li/Ge one, and (**c**) the first Brillouin zone.

The ternary compound, Li_2_GeO_3_ with 24 atoms within a conventional unit cell, is chosen for a model study in illustrating the geometric, electronic and optical properties. The optimal lattice, as clearly shown in Fig. 1 and table S1, belongs to an orthorhombic structure. Each Li/Ge atom is surrounded by four O atoms in a tetrahedral form. There exist 32 Li-/16 Ge–O bonds, in which the former and the latter display large fluctuations about their lengths (∼1.952–2.167 Å and ∼1.757–1.870 Å, respectively). The calculated lattice constants along the x-, y- and z-directions, (9.612, 5.462, 4.874 Å), are very close to the X-ray diffraction measurements, (9.602, 5.502, 4.849 Å) in Ref. 5 and (9.632, 5.479, 4.842 Å) in Ref. 22. Obviously, the greatly non-uniform chemical/physical environment indicates the importance of orbital hybridizations in chemical bondings. Furthermore, it might be very useful for supporting the ion transport in electrolyte/electrode materials^14^. This behavior will be responsible for the unusual electronic properties and the highly anisotropic optical transitions.

**Figure 2:**
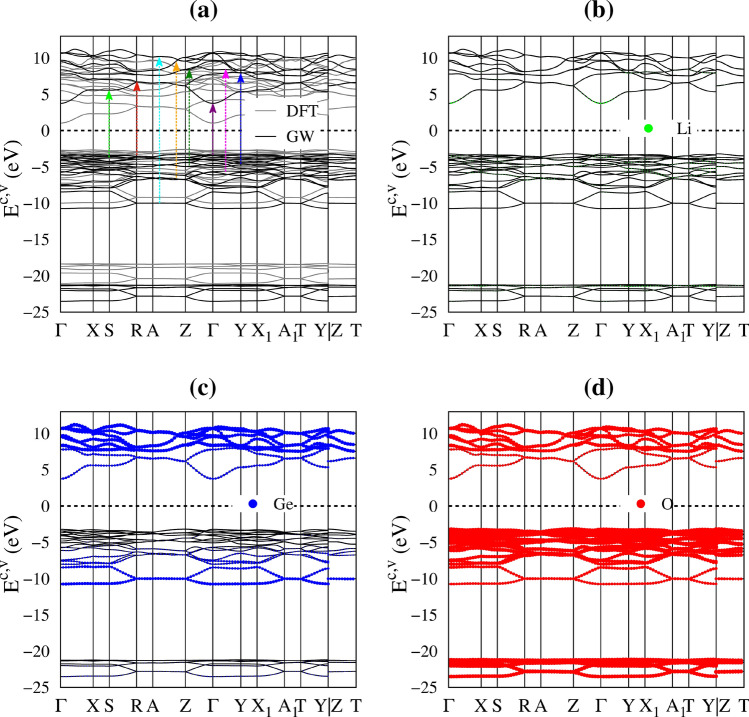
(**a**) The quasi-particle band structure along the high-symmetry points in the wave-vector space (solid-black lines), the standard DFT calculation (the solid-grey lines) is also added for comparisons, the vertical-colored arrows indicate the optical excitations. The domination of (**b**) Li, (**c**) Ge and (**d**) O atoms (the green, blue and red circles) in the electronic band structure. The Fermi level is denoted by the dash-black line.

Li_2_GeO_3_ exhibits a rich and unique band structure. The main features in Fig. 2a show a lot of valence and conduction sub-bands in a wide energy range (− 26 eV < E^c;v^ < 12 eV), the high asymmetry of hole and electron energy spectra about the Fermi level (E_F_ = 0), the parabolic/oscillatory/linear/partially flat energy dispersions with various band-edge states, and the atom-dominated electronic states are frequently present in the different energy regions. An indirect band gap of $E_{g}^{i} = 6.9 eV$ related to the highest occupied state and the lowest unoccupied one, respectively, at the Z and Γ points. The quasi-particle band gap in this work is systematically larger than the corresponding standard DFT band gap (the grey curves with the energy gap of 3.8 eV)^6^, which suggest an enhancement of electron–electron interactions^20^ (The detail explanations as shown in supporting information). In addition, the low screening leads to excitonic effects well below the quasiparticle band gap. This point will be discussed in the next section.

The electronic structures can be classified into five categories based on the various atom and orbital contributions, as clearly indicated in Fig. 2b–d by the green, blue and red circles for Li, Ge and O, respectively. The (I), (II), (III), (IV) and (V) regions, respectively, correspond to E^c^ > 7.6 eV, 3.2 eV < E^c^ < 7.6 eV, − 6.0 eV < E^v^ < − 3.8 eV, − 10.6 eV < E^v^ < − 6.0 eV, and − 24.5 eV < E^v^ < − 21.8 eV. It should be noted that valence sub-bands disappear between the (IV) and (V) regions. The Li atoms only make very weak, yet still significant contributions within a whole range of band structures (small green circles in Fig. 2b), i.e., the unusual essential properties disappear in the absence of Li–O bonds. The energy-spectrum sub-groups are qualitatively characterized by (I) (Ge, O) co-dominance, (II) (Ge, O) co-dominance, (III) O dominance, (IV) (Ge, O) co-dominance and (V) O dominance, being supported by the atom and orbital-projected density of states (discussed later in Fig. 4). The specific orbital hybridizations in chemical bonds will be identified to be associated with the critical energy bands in revealing the prominent absorption structures.

**Figure 3:**
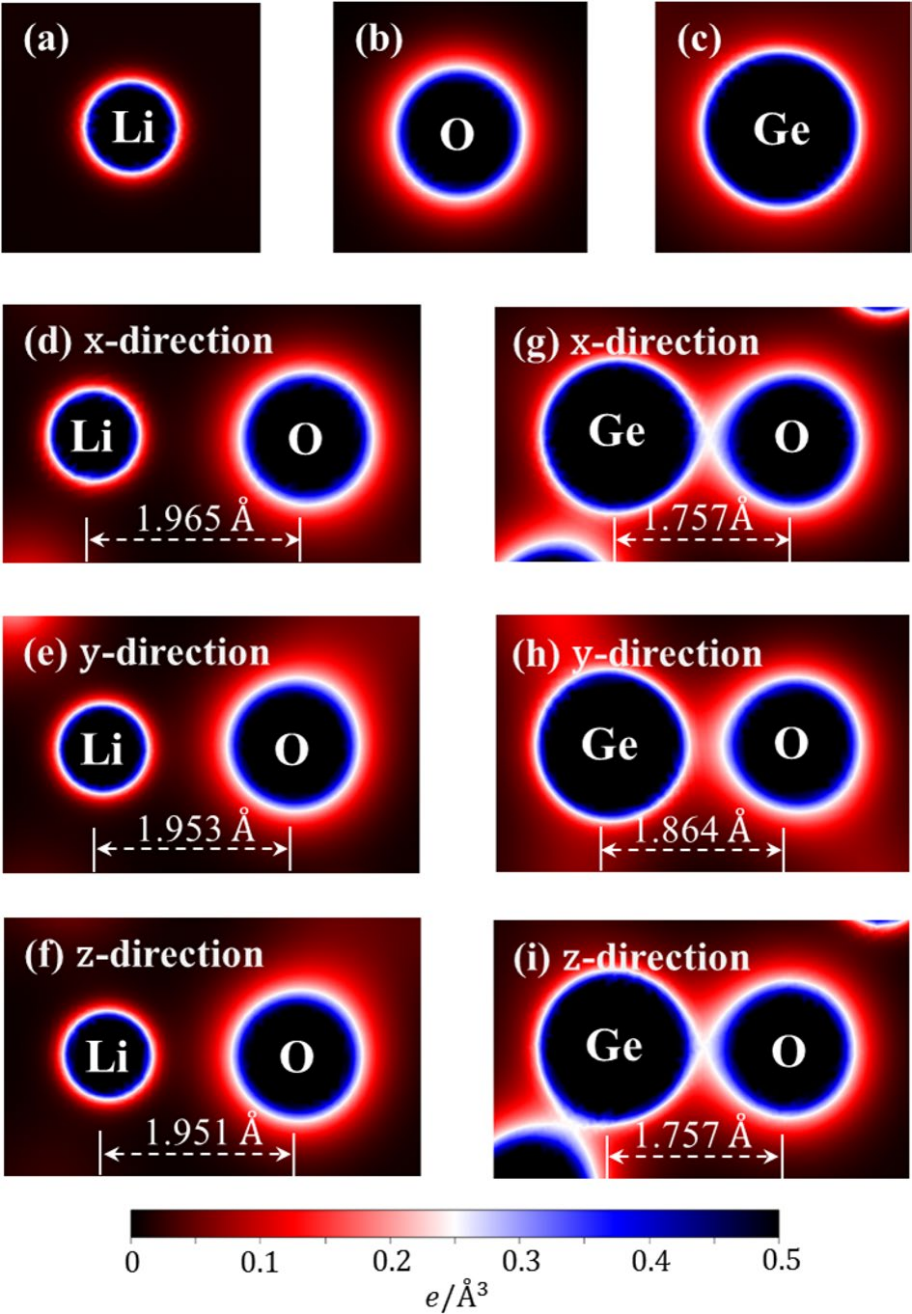
The spatial charge density distributions for (**a**)/(**b**)/(**c**) an isolated Li/Ge/O atom and (**d**)/(**e**)/(**f**) the shortest Li–O & (**g**)/(**h**)/(**i**) Ge–O bond along x-/y-/z-direction, respectively.

The spatial charge distributions before/after chemical bonding can provide some very useful information about the first-step orbital hybridizations in Li–O and Ge–O bonds. An isolated Li atom has an isotropic charge density (Fig. 3a), in which the inner and outer regions (the dark-blue and white-red parts) arise from 1 and 2s orbitals, respectively. The similar, but wider distribution, which corresponds to the O case (Fig. 3b), is associated with (1s, 2s) and (2p_x_, 2p_y_, 2p_z_) orbitals. Furthermore, the highest charge density appears around Ge (Fig. 3c), with the separate ranges of (4s, those below it) and (4p_x_, 4p_y_, 4p_z_) orbitals. As for the Li–O bonds, the outer/inner regions show the obvious/minor deformations along three electric-polarization directions (Fig. 3d–f), especially for the neighboring ones. These clearly indicate the multi-orbital hybridizations of 2s–(2p_x_, 2p_y_, 2p_z_)/the single-orbital hybridization of 2s–2s. In addition to the white-red regions (Fig. 3g–i), the dark-blue ones near the Ge and O atoms present observable changes, dense charge density between Ge and O atoms and short Ge–O bonds suggest the significant ionic-covalent bonding of (4s, 4p_x_, 4p_y_, 4p_z_)–(2s, 2p_x_, 2p_y_, 2p_z_). To further quantitatively analyze the bonding character, the Bader charge analysis of the Li_2_GeO_3_ compound were also performed. The average effective charges are determined to be 0.88 e for Li atom, 2.13 e for Ge atom and − 1.3 e for O atom. This indicates that Li atoms and Ge atoms transfer their electrons to O atoms to form a stable structure. Very interestingly, the Li/Ge atom give almost all/haft of its valence charges and therefore, Li–O/Ge–O bonds exhibit the ionic/mixture of ionic and covalent characteristics.

**Figure 4:**
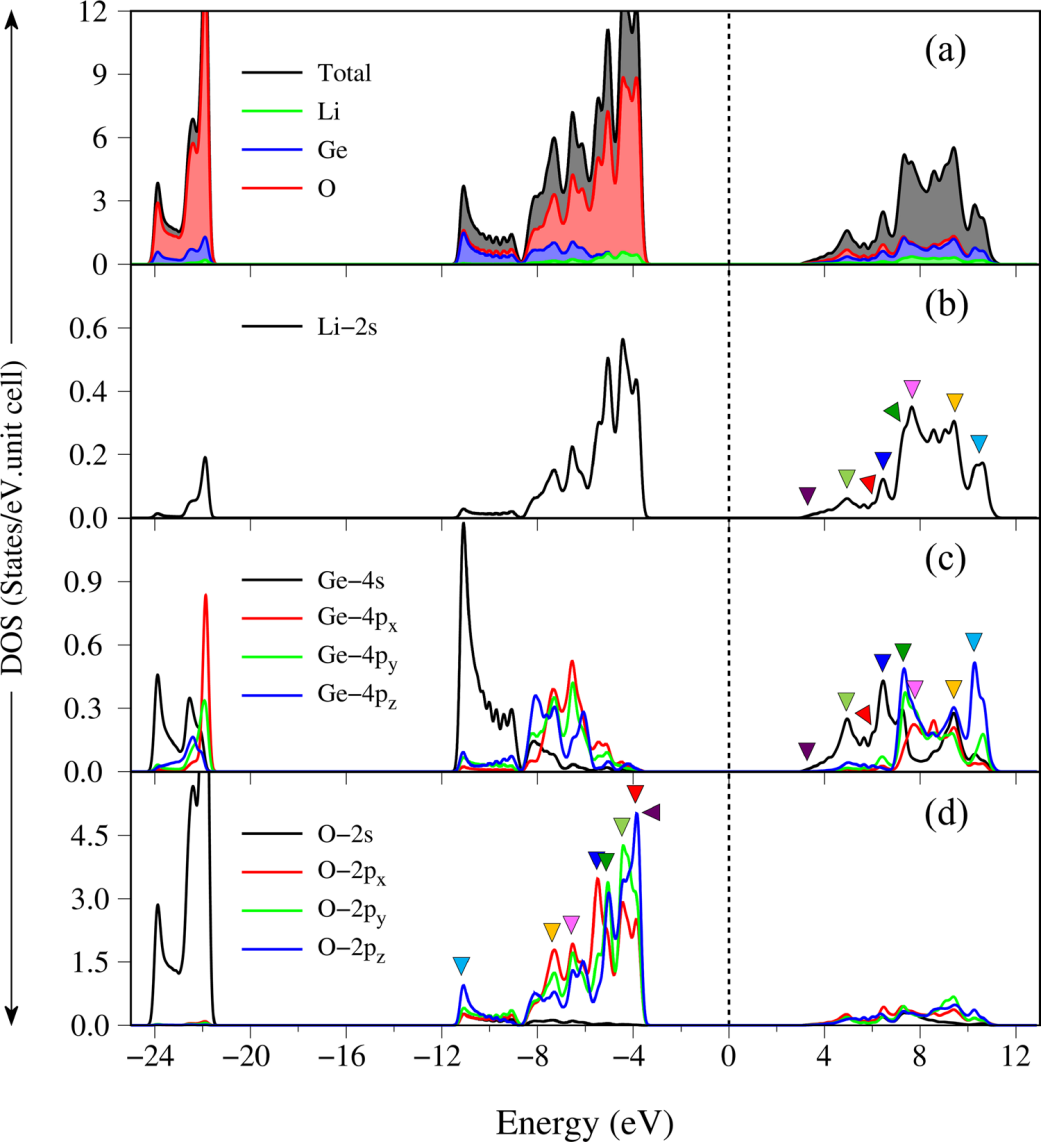
The G_0_W_0_ quasi-particle density of states for different components: (**a**) total magnitude with Li-, Ge-, O-atom contributions, (**b**) Li-2s orbitals, (**c**) Ge-(4s, 4p_x_, 4p_y_, 4p_z_) orbitals, and (**d**) O-(2s, 2p_x_, 2p_y_, 2p_z_) orbitals. The colored triangles indicate the corresponding optical excitations, the dash line represents the Fermi level.

The atom and orbital-projected density of states (DOS), being supported by the atom-dominated band structure (Fig. 2) and charge density distribution (Fig. 3), can clearly identify the energy-dependent orbital hybridizations. Their van Hove singularities—the special structures in DOS originated from the critical points in the energy band structures, are mainly governed by the energy dispersion in the energy-wave-vector spaces and dimensionality^23,24^. As a result of many band-edge states with various energy dispersions, such as the extreme, saddle and dispersionless ones, Fig. 4a displays various prominent asymmetric peaks and shoulders (the black curves). The van Hove singularities of different atoms and orbitals can merge (Fig. 4a–d), clearly indicating the specific orbital hybridizations. According to their strong co-relations, the five subgroups of band structure are further examined to be dominant through (I) 2s–(2s, 2p_x_, 2p_y_, 2p_z_) & (4s, 4p_x_, 4p_y_, 4p_z_)–(2s, 2p_x_, 2p_y_, 2p_z_), (II) 2s–(2p_x_, 2p_y_, 2p_z_) & 4s–(2p_x_, 2p_y_, 2p_z_), (III) 2s–(2p_x_, 2p_y_, 2p_z_) & (4s, 4p_x_, 4p_y_, 4p_z_)–(2p_x_, 2p_y_, 2p_z_), (IV) 2s–(2p_x_, 2p_y_, 2p_z_) & 4s–(2p_x_, 2p_y_, 2p_z_) and (V) the single orbital 2s–2s & 4s–2s. Such identifications of significant orbital bonding would become more delicate under strong optical responses.

After the perturbation of an electromagnetic (EM) wave, electrons are vertically excited from the occupied to unoccupied states. The excited valence holes and conduction electrons simultaneously come into existence during the optical excitations; furthermore, they tend to combine through the attractive Coulomb potentials under the suitable condition, e.g., a large band gap for the suppression of temperature broadening. The coupled quasiparticles, the stable excitons, might strongly affect the main features of the optical absorption spectra since they make important contributions to many-body effects. This mean that they take part in the various-order excitation processes using the Coulomb scatterings, leading to the dramatic transformations of photons. The excitonic effects, which are closely related to the critical orbital hybridizations, are the focus of this study.

**Figure 5:**
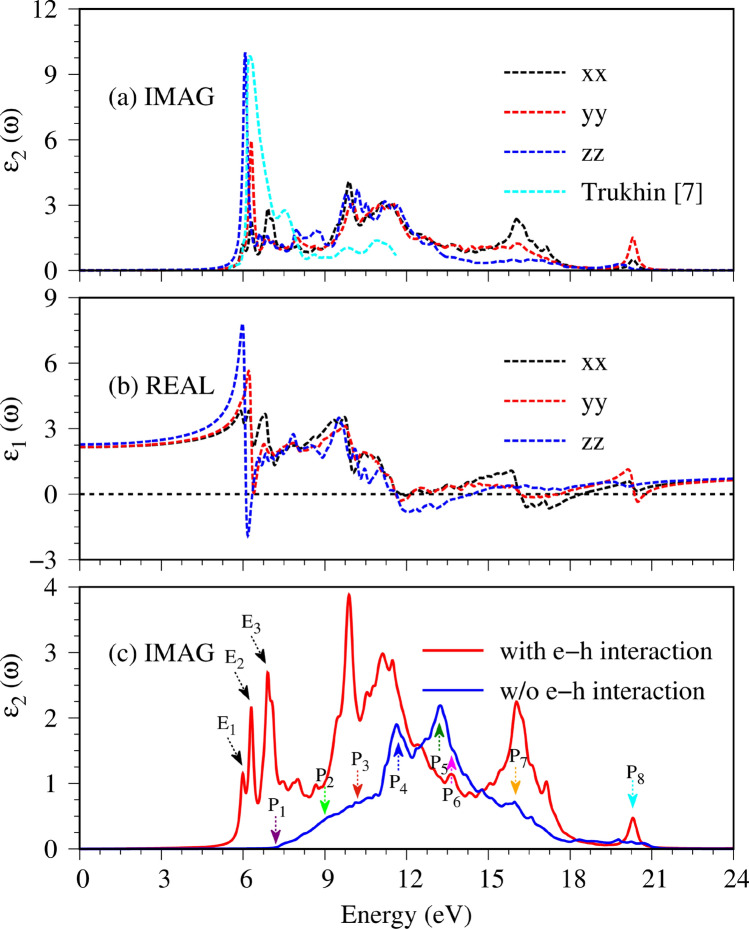
(**a**) The imaginary and (**b**) real parts of dielectric functions with the excitonic effects under three electronic-polarization directions (The dash black, red and blue lines). The previous experiments result (the dash cyan line) is also added. (**c**) Comparison of the imaginary parts of the dielectric function of x-direction with and without excitonic effects. The independent-particle optical absorption and the three first exciton peaks are, respectively, made with colored and black arrows.

As a consequence of the electromagnetic wave absorption, the dielectric function is complex and reflects the main features of electronic properties and thus orbital-hybridizations. The imaginary part, $\epsilon_{2} \left( \omega \right)$, as clearly showed in Fig. 5a, presents the excitation characteristics of available channels in the absence of a screening effect of all valence charges. Because of the non-uniform environment in the orthorhombic structures, the optical absorption of Li_2_GeO_3_ is three-fold degenerate and manifests a strong polarization effect. An optical gap ($E_{g}^{o}$), which corresponds to the threshold absorption frequency, is about 5.95/5.92/5.87 eV under the many-body effects for the x-/y-/z-directions of electric polarizations (the black/red/blue curves). However, it is ∼ 7.00 eV purely through the single-particle optical excitations for ${\varvec{E}}\parallel {\varvec{x}}$ (the blue curve in Fig. 5c). Compared with band gap of $E_{g}^{i}$ = 6.9 eV, the great red shift clearly indicates the very strong Coulomb couplings between the excited holes and electrons. The composite quasiparticles should be quite stable, so that they are expected to survive at room temperature. The theoretical prediction is relatively in good accord with the previous experiment result (the cyan curve in Fig. 5a)^7^.

**Figure 6:**
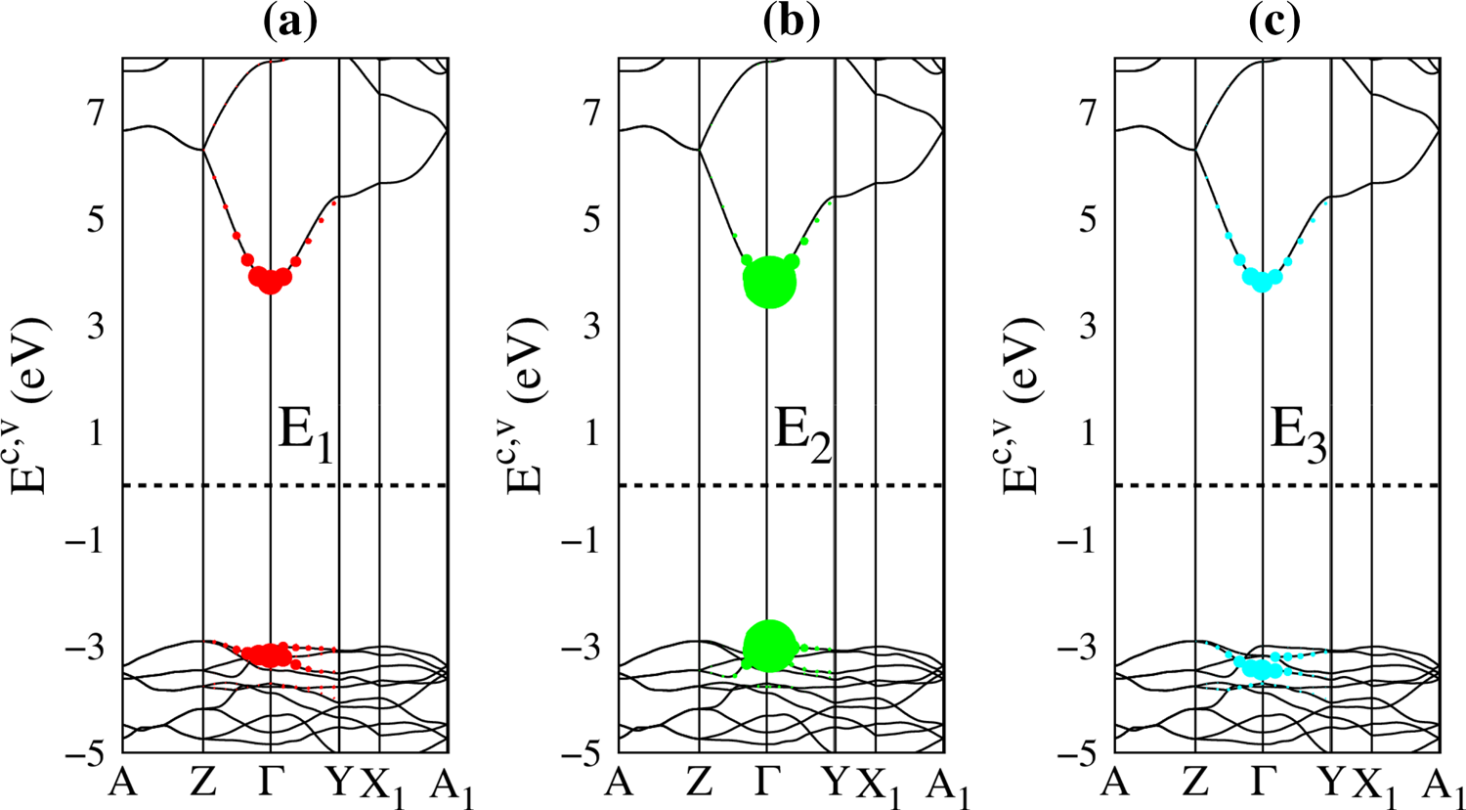
The amplitude of (**a**) the first, (**b**) second and (**c**) third excitons are plotted as fat-band styles. The radii of circles represent the contribution of electron–hole pair at that k-point to the *i*th exciton wave function, the solid black lines in the background are the corresponding G_0_W_0_ quasi-particle band structures, the dash line represents the Fermi level.

In addition to the optical gap, the main profile of the single particle optical excitations also exists various pronounced peaks or shoulder structures (the distinct colored arrows in Fig. 5c). In which, the appearance of these certain optical spectral structures is associated with the transitions from band-edge states/Van Hove singularities of electronic band structure/orbital-projected density of states. Furthermore, the formation of the robust excitonic states around the respective extrema of the band-edge state also creates extra prominence peaks below the band gap (the black arrows in Fig. 5c) and largely modify optical transition intensities. The frequency-dependent vertical transitions and orbital hybridizations are identified (detail in Table 1), the corresponding excitation of each channel is marked with arrow heads in Fig. 2a and the colored triangles in Fig. 4. For example, the excitation from the valence flat band to the conduction minimum extremes at the Γ point, leading to the threshold structure of the independent excitation spectrum (purple arrow in Fig. 5c). The shoulder structures, which arise from the several extreme-related transitions with equivalent frequency (light-green, red and cyan arrows in Fig. 5c as examples), while the sharp peaks (blue and green arrows) correspond to the optical excitations between flat bands, for which, their inequivalent intensities are associated with the relevant hybridizations. Furthermore, the orbital character of three lowest excitons is also described as fat bands—the reciprocal-space distribution of excitons^25,26^. As shown in Fig. 6, the contributions to these most intense excitons arise mostly from the band extrema vicinity of the Γ point. The three highest occupied and one lowest unoccupied state, where most of the excitonic weight is localized, are characterized by O-(2p_x_,2p_y_,2p_z_) and Ge-(4s) orbitals hybridizations, respectively. Additionally, due to increasing electron–hole overlaps, the absorption coefficient above the band gap is significantly enhanced. Using this strategy, the specified mechanisms for the various absorption structures could be achieved. This viewpoint has been successfully generalized to other emergence materials.

**Table 1:** Calculated the prominent absorption structures and the leading transition of each peaks. E_*i*_ denotes *i*th excition, while P_*i*_ denotes *i*th interband transition peaks.

Peaks	Colors	Energy (eV)	Specific orbital hybridizations in Ge–O bonds
E_1_	Black	6.0	Ge (4s) – O (2p_x_,2p_y_,2p_z_)
E_2_	Black	6.3	Ge (4s) – O (2p_x_,2p_y_,2p_z_)
E_3_	Black	6.9	Ge (4s) – O (2p_x_,2p_y_,2p_z_)
P_1_	Purple	7.0	Ge (4s) – O (2p_x_,2p_y_,2p_z_)
P_2_	Ligh-Green	9.0	Ge (4s) – O (2p_x_,2p_y_ 2p_z_)
P_3_	Red	10.2	Ge (4s) – O (2p_x_,2p_y_ 2p_z_)
P_4_	Blue	11.4	Ge (4s) – O (2p_x_,2p_y_ 2p_z_)
P_5_	Green	13.0	Ge (4p_x_, 4p_y_, 4p_z_) – O (2p_x_,2p_y_ 2p_z_)
P_6_	Purple	14.0	Ge (4p_x_, 4p_y_, 4p_z_) – O (2p_x_,2p_y_ 2p_z_)
P_7_	Yellow	15.8	Ge (4p_x_, 4p_y_, 4p_z_) – O (2p_x_,2p_y_ 2p_z_)
P_8_	Cyan	20.3	Ge (4p_x_, 4p_y_, 4p_z_) – O (2p_x_,2p_y_ 2p_z_)

**Figure 7:**
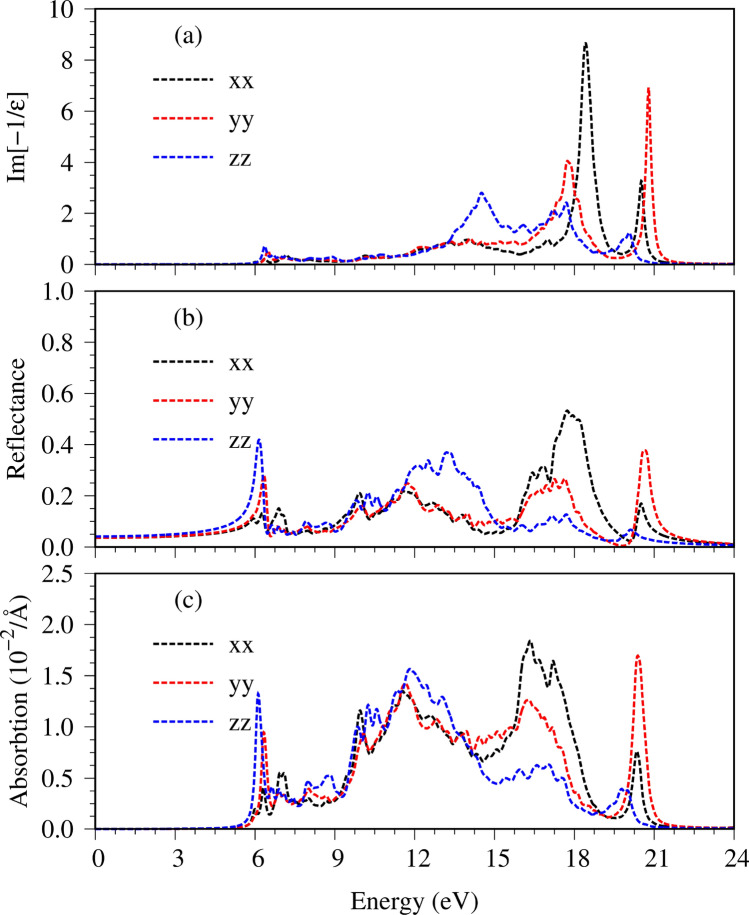
The various optical properties: (**a**) energy loss functions, (**b**) reflectance spectra, and (**c**) absorption coefficients.

For the real part $\epsilon_{1} \left( \omega \right)$ (Fig. 5b), their prominent absorption structures can be understood from those of $\epsilon_{2} \left( \omega \right)$ by the Kramers–Kronig relation $\left( \epsilon {_{1} \left( \omega \right) = 1 + \frac{2}{\pi }P\mathop \smallint \limits_{0}^{\infty } \frac{{\omega^{^{\prime}2}\epsilon_{2}\left( \omega \right)}}{{\omega^{^{\prime}2} - \omega^{2} }}d\omega^{\prime}} \right)$ under the event of optical excitations^27^. Apparently, the special structures in $\epsilon_{1} \left( \omega \right)$ and $\epsilon_{2} \left( \omega \right)$ might be similar or different, being strongly co-related by the principal-value integration on the complex plane. Below the threshold frequency,$\epsilon _{1} \left( \omega \right)$ weakly depends on $\omega$, in which its value roughly lies within the range of 2.1–2.2 for x-, y-polarizations and the z one. This will determine the low-frequency reflectance spectrum (Fig. 7b) and the vanishing absorption coefficient (Fig. 7c). Very interesting, $\epsilon_{1} \left( \omega \right)$ is very sensitive to the changes of frequency during the creation of the excited holes and electrons. It can vanish at weak Landau damping, in which its zero point and the small $\epsilon_{2} \left( \omega \right)$ might appear simultaneously, e.g., the vanishing $\epsilon_{1} \left( \omega \right)$ at 18.5/20/13.6 eV under ${\varvec{E}}\parallel {\varvec{x}}/{\varvec{E}}\parallel {\varvec{y}}/{\varvec{E}}\parallel {\varvec{z}}$. In addition, the zero points for the z-polarization (the blue curve) at 6 eV become meaningless because of the combination with very strong electron–hole excitations. The other interesting phenomena occurring in our calculation is the presence of the isotropy in the high-frequency dielectric tensor ($\epsilon_{1} \left( \infty \right)$ = 0.77/0.77/0.83 for x-/y-/z-directions), which may have good optical properties, and has far-reaching research signification.

The energy loss function (ELF), being defined as $Im\left[ {1/ \epsilon \left(\omega \right)} \right]$ is the screened response function due to the significant valence charges of the Li, Ge and O atoms. The charge screenings can determine the coherent carrier oscillations at the long wave-length limit during the optical transitions. In general, the collective excitations (a plasmon mode) are revealed as a sufficiently strong peak with ELF higher than 1, as clearly indicated in Fig. 7a. The strongest peak comes into existence at $\omega_{P}$ = 18.5/20/13.6 eV for the x-/y-/z-direction electric polarizations, being attributed to the significant Li–2s, O–(2p_x_, 2p_y_, 2p_z_) & Ge–(4s, 4p_x_, 4p_y_, 4p_z_) orbitals. The O–2s contribution for the most prominent plasma wave is ignored, since its dominating valence states only appear below − 22 eV (the black curve in Fig. 4d). In addition, a few minor plasmon peaks are accompanied by serious Landau dampings. It should be noted that two manners could be utilized to identify the collective excitations, but the current peak in ELF is much better than the zero points in $\epsilon_{1} \left( \omega \right)$. The absence of the latter and/or the combination with a very large $\epsilon_{2} \left( \omega \right)$ is the main reason.

When an electromagnetic wave is normally incident on Li_2_GeO_3_. The total electric field will be reflected by the surface, absorbed by the valence electrons and transmitted through a finite-width sample. The reflectance $R\left( \omega \right)\, \left(\mathrm{which\,is\,characterized\,as}\,\left| {\sqrt {\frac{\epsilon\left( \omega \right) - 1}{{\epsilon\left( \omega \right) + 1}}} } \right|^{2}\right)$ and the absorption coefficient $\alpha \left( \omega \right)\,\left(\mathrm{defined\,as}\,\sqrt 2 \omega \left[ {\sqrt {\epsilon _{1}^{2} \left( \omega \right) +\epsilon _{2}^{2} \left( \omega \right)} -\epsilon_{1} \left( \omega \right)} \right]^{1/2} \right)$ are two characteristic optical phenomena of solid-state materials, directly reflecting the main features of the single-particle (Fig. 5a,b) and the collective excitations (Fig. 7a). As shown in Fig. 7b,c, for $\omega < E_{g}^{o}$, a small reflectance weakly depends on the frequency and is roughly given by $\left| {\sqrt {\frac{{\epsilon _{1} \left( 0 \right) - 1}}{{\epsilon _{1} \left( 0 \right) + 1}}} } \right|^{2} .$ The absorption coefficient $\alpha \left( \omega \right) = 0$ because of the vanishing electron–hole excitations, leading to the non-decay EM-wave propagation and thus a very efficient transmission. However, within the active region of valence-electron excitations $\left( {E_{g}^{o} < \omega < 21 eV} \right)$, the sensitive and significant frequency dependences come into existence. Reflectance is enhanced and displays a large fluctuation under the various inter-band excitation channels, in which a drastic change of the plasmon edge appears at $\omega_{P}$, e.g., ∼ 40% variation under the ${\varvec{E}}\parallel {\varvec{y}}$ case (the red curve). Moreover, since absorption coefficient $\alpha \left( \omega \right)$ is very large, especially that related to the plasmon mode, and its inverse is about 50–300 Å, the EM waves are easily absorbed by Li_2_GeO_3_ through the rich electronic excitations.

In addition to X-ray diffractions, only very few experimental examinations on electronic and optical properties exist. In general, the wave-vector dependences of occupied valence states can be directly tested by angle-resolved photoemission spectroscopy (ARPES)^28^. A lot of unusual energy sub-bands (Fig. 2) due to a large and complex unit cell would create high barriers in the ARPES measurements. Scanning tunneling spectroscopy (STS)^29^ is available for the clear identification of van Hove singularities near the Fermi level. However, STS might be suitable only under a thin-film sample because of very weak quantum currents. Very interestingly, the optical spectroscopy methods of reflectance^30^, absorption^31^ and transmission^32^ are reliable in verifying the frequency-dependent optical properties. They can provide significant information about the initial excitonic peaks, the greatly reduced threshold excitation frequency, many prominent absorption structures, and the strongest plasmon mode at $\omega_{P}$ = 6.0 eV. Up to now, one photo-luminescence measurement has verified the optical gap situated at ~ 6.0 eV^7^, being close to the current prediction of ~ 5.95 eV. Apparently, the diverse optical properties in lithium-based compounds are worthy of systematic investigations, both experimentally and theoretically.

## Conclusion

The significant orbital hybridizations in chemical bonds, being based on first-principles calculations, are thoroughly identified for the Li_2_GeO_3_ compound in terms of the geometric, electronic and optical properties. They will play important roles in fully understanding the diversified essential properties of optical/electrode materials in ion-related materials. This material presents unusual features, i.e. a large unit cell with a highly non-uniform chemical environment, the Li-, Ge- or O-dominated energy bands, orbital-induced spatial charge densities, atom- and orbital-decomposed van Hove singularities. As a result, the band-edge states, which might create the prominent optical responses, are well characterized by the specific orbital interactions through the developed theoretical framework. The featured optical transitions cover a red-shift optical gap ($E_{g}^{o}$ = 5.95 eV) much lower than an indirect one ($E_{g}^{i}$ = 6.9 eV), a very long transmission length/low reflectance for $\omega < E_{g}^{o}$, various pronounced single-particle absorption structures (short decay lengths), and the strongest plasmon peak/a quickly decreasing edge in the energy loss function/reflectance spectrum at $\omega_{P}$ = 17–18 eV, and sensitive changes due to the electric-field directions. The many-body excitonic effects have strongly modified the single-particle inter-band excitations. Under current investigations, the developed theoretical framework can be further generalized to include other emergence materials.

## Supplementary Information

Supplementary Information.
