# Supplementary material for: Orbital-hybridization-created optical excitations in Li2GeO3
Source: Sci Rep. 2021 Mar 2;11:4939. doi: 10.1038/s41598-021-84506-0 (PMC7925650; doi:10.1038/s41598-021-84506-0)
Supplement: Supplementary file 1 — Supplementary Information. [file 41598_2021_84506_MOESM1_ESM.docx]

**Orbital-hybridization-created optical excitations in Li_2_GeO_3_**

Vo Khuong Dien^1*^, Hai Duong Pham^2^, Ngoc Thanh Thuy Tran^3^, Nguyen Thi Han^1^, Thi My Duyen Huynh^1^, Thi Dieu Hien Nguyen^1,*^, and Ming Fa-Lin^1,3,*^.

^1^Department of Physics, National Cheng Kung University, Tainan 701, Taiwan,

^2^Center of General Studies, National Kaohsiung University of Science and Technology, Kaohsiung, Taiwan,

^3^Hierarchical Green-Energy Materials (Hi-GEM) Research Center, National Cheng Kung University, Tainan 701, Taiwan.

[*vokhuongdien@gmail.com, nguyenhien1901@gmail.com](mailto:*vokhuongdien@gmail.com,%20nguyenhien1901@gmail.com) and [mflin@mail.ncku.edu.tw](mailto:mflin@mail.ncku.edu.tw)

Table S1. Structure parameter of 3D ternary Li_2_GeO_3_ compound.

|  | Lattice constants | | | Volume $\left( Å^{3} \right)$ | Bond length $\left( Å \right)$ | |
| --- | --- | --- | --- | --- | --- | --- |
|  | a $\left( Å \right)$ | b $\left( Å \right)$ | c $\left( Å \right)$ |  | Li-O | Ge-O |
| This work (PBE) | 9.612 | 5.462 | 4.874 | 255.889 | 1.951 (8)  1.953 (8)  1.965 (8)  2.168 (8) | 1.757 (8)  1.863 (4)  1.864 (4) |
| This work (LDA) | 9.083 | 5.148 | 4.600 | 215.093 | 1.794 (8)  1.803 (8)  1.822 (8)  1.926 (8) | 1.692 (8)  1.794 (4)  1.802 (4) |
| X-ray diffraction ^1^ | 9.632 | 5.479 | 4.842 | 255.530 | - | - |
| X-ray diffraction ^2^ | 9.602 | 5.502 | 4.849 | 256.173 | - | - |

The structure and ground-state properties of Li_2_GeO_3_ have been investigated within the density functional approximations. In our calculation, by adopted different computational techniques, the ground state properties and the optimization structure have been achieved. The equilibrium crystal structure and the calculated lattice parameter are listed in Table S1, with other available experimental data. The lattice constants obtained from PBE approximation are much closer to experimental results than for those obtained by LDA approach. Therefore, we select the most suitable method, GGA-PBE functional, for further calculations.


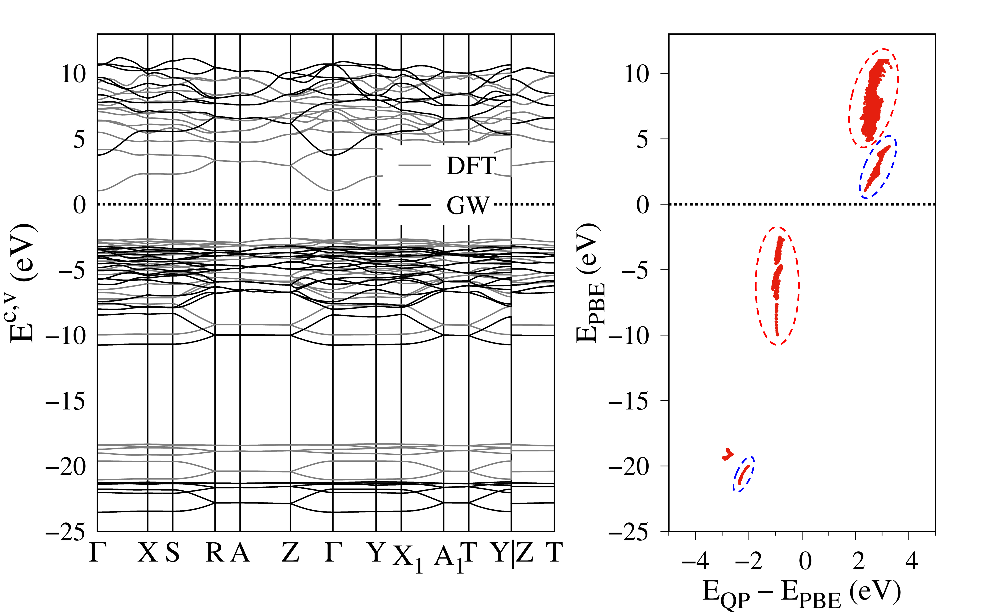


Figure S1: The quasi-particle and the standard DFT band structures (left panel) are described by the solid black and solid grey lines, respectively. The differences between the GW quasi-particle energy and PBE Kohn-Sham eigenvalue (right panel) of a Li_2_GeO_3_ compound. The Fermi level (dash-line) is set to be zero.

The GW self-energy corrections to the DFT band structure for the Li2GeO3 compound are shown in Fig. S1. After taking the e-e self-energy effects into account, the valence and conduction states are strongly modified and the band gap increases from 3.8 eV to 6.9 eV. Such large changes are due to the strong ionic-covalent bonds, a dense spatial charge density which leading the large quasi-particle corrections and thus, enhanced the electron-electron interactions ^3-6^. In addition, the quasiparticle energy corrections also show a complicated orbital character and energy dependence, for example, the quasi-particle energy correction to s-orbitals (the dash-blue oval in Fig. S1) are linear change, while those to p-orbitals (the dash-red oval in Fig. S1) are almost constant. The energy band in the valence bands and conduction bands also behave differently. As a result, the quasi-particle band structure cannot be described by a simple scissor operator.

**References**

1. Rahman, M.M. et al. Lithium germanate (Li2GeO3): a high‐performance anode material for lithium‐ion batteries. *Angewandte Chemie* **128**, 16293-16297 (2016).

2. Yin, C., Xiang, H., Li, C., Porwal, H. & Fang, L. Low-temperature sintering and thermal stability of Li2GeO3-based microwave dielectric ceramics with low permittivity. *Journal of the American Ceramic Society* **101**, 4608-4614 (2018).

3. Liang, Y. & Yang, L. Electronic Structure and Optical Absorption of Fluorographene. *MRS Proceedings* **1370**, mrss11-1370-yy1304-1302 (2011).

4. Aggoune, W., Rezouali, K. & Belkhir, M. A. Strong excitonic effects in hydrogen–graphene–fluorine janus graphene. *physica status solidi (b)* **253**, 712-717 (2016).

5. Thatribud, A. Electronic and optical properties of TiO2 by first-principle calculation (DFT-GW and BSE). *Materials Research Express* **6**, 095021 (2019).

6. Wei, W. & Jacob, T. Electronic and optical properties of fluorinated graphene: A many-body perturbation theory study. *PhRvB* **87**, 115431 (2013).
